# Editorial: The Effect of Herbal Medicine on Intestinal Flora and the Potential Molecular Mechanism

**DOI:** 10.3389/fphar.2023.1195102

**Published:** 2023-06-12

**Authors:** Wei Liu, Xueyang Deng, Wenyi Kang, Jing Qian, Esra Küpeli Akkol, Alessandra Durazzo, Massimo Lucarini

**Affiliations:** ^1^ Institute of Plant Protection and Microbiology, Zhejiang Academy of Agricultural Sciences, Hangzhou, China; ^2^ School of Traditional Chinese Pharmacy, China Pharmaceutical University, Nanjing, China; ^3^ National Research and Development Center for Edible Fungus Processing Technology, Henan University, Kaifeng, China; ^4^ Henan Province Food Engineering Technology Research Center, Kaifeng, China; ^5^ Pharmaceutical Informatics Institute, College of Pharmaceutical Sciences, Zhejiang University, Hangzhou, China; ^6^ Department of Pharmacognosy, Faculty of Pharmacy, Gazi University, Ankara, Türkiye; ^7^ CREA-Research Centre for Food and Nutrition, Rome, Italy

**Keywords:** herbal medicine, intestinal flora, molecular mechanism, physiological function, infection

Herbal medicine has been used for centuries to treat a variety of ailments, and recent research has shown that it can have a significant impact on intestinal flora. Studies have shown that after oral administration of herbal medicine, the structure and metabolism of intestinal flora can be regulated.

In this Research Topic, the effect of herbal medicine on intestinal flora and the potential molecular mechanisms, as well as the potential therapeutic benefits of herbal medicine on various diseases, was explored. The understanding of the potential function of plants is developing rapidly, also at the interface of food and medicine (Akkol et al., 2020; Durazzo et al., 2022). These functional herbal medicines have demonstrated anti-inflammatory, anti-tumor, antimicrobial, antioxidant, and/or antiaging actions related to their bioactive components (Ali et al., 2022; Kupeli Akkol et al., 2022; Yucel et al., 2022; Ye et al., 2023).

Nowadays, gut microbiota deviations are linked with many diseases and herbal medicine is considered for their treatment. The intestinal microbiota was reported to regulate the progress of sepsis and attenuate organ damage. Traditional Chinese medicine (TCM) was introduced to prevent the progress of sepsis and improve the prognosis of patients with sepsis by improving the imbalance of intestinal microbiota, improving immunity and reducing the damage to the intestinal barrier (Wang X-H. et al.). Xuebijing has been frequently used for treating sepsis, and its beneficial effect against *Pseudomonas aeruginosa* infection in *Caenorhabditis elegans* was elegantly elucidated (Zhang et al.). Herbal medicine also has unique advantages in the treatment of female reproductive disorders. The mechanisms of TCM in the treatment of female reproductive disorders were reviewed with the hope for the development of new herbal medicines (Liu et al.). Sodium cantharidate, a derivative of cantharidin, was shown to promote autophagy in breast cancer cells by inhibiting the Pl3K-Akt-mTOR signaling pathway (Pang et al.).

The intestinal flora plays a crucial role in maintaining the host’s physiological functions. A disruption of the fragile host-microbiota interaction equilibrium underlies the pathogenesis of many disorders. Niuhuang Pill was shown to ameliorate cerebral ischemia/reperfusion injury in mice partly by restoring gut microbiota dysbiosis, such as the phyla Bacteroidetes and Firmicutes, the families Lachnospiraceae and Prevotellaceae, and the genera Alloprevotella and Roseburia (Zhang et al.) As the animal experiment by Li C et al. revealed that Qiweibaizhu powder crude polysaccharide could further treat antibiotic-associated diarrhea by restoring the diversity, relative abundance and community structure of intestinal mucosal microbiota, which reveals the mechanism of herbal medicine polysaccharide in the treatment of diarrhea disorders (Li C et al.). Gut fungi differentially response to the antipyretic (heat-clearing) and diaphoretic (exterior-releasing) TCM in Coptis chinensis-conditioned gut microbiota. Their analysis revealed that the diaphoretic TCMs-enriched fungi Fusarium spp. were positively related to Akkermansia spp., a beneficial bacterium that interacts with Toll-like receptor (Yang et al.). As a study by Li X. et al. revealed that Dendrobium officinale (DO) had a moderating effect on diversity, community structure, and functions of intestinal contents microbiota in mice fed with high-fat diet. They speculated that the mechanism of DO against high-fat diet diseases might be attributed to the inhibition of Ruminococcus and Oscillospira, leading to a promotion in the state of host health. Polysaccharides are commonly found in most herbal medicines and have important medicinal value (Li et al.).

Besides the intestinal microbiota plays an important role in maintaining intestinal health, gut microbiota-derived metabolites are closely associated with the development of disease. A study presented in the issue links high-salt diet and hypertension through intestinal microbiota, metabolites and metabolic pathways, providing new insights into the microbial mechanism of high-salt diet-induced hypertension. The results showed that a high salt diet promoted hypertension *via* the inhibition of Clostridiaceae_1 growth and alterations in the GABA metabolic pathway, leading to increased blood pressure (Zheng et al.). Another study showed that Wine-processed Radix Scutellariae (WRS) ameliorated SEB-induced ARDS by regulating the structure of gut microbiota, increasing the production of SCFAs and modifying the faecal metabolite profiles through the lung-gut axis (Hu et al.). Also, *Cordyceps guangdongensis* lipid-lowering formula was reported to alleviate fat and lipid accumulation by modulating gut microbiota and short-chain fatty acids in high-fat diet mice (Wang et al.). In addition, wheat supplement with buckwheat affect gut microbiome composition and circulate short-chain fatty acids (Yao et al.).

In sum, herbal medicine has been used for centuries to treat a variety of ailments. Recently, research on this Research Topic: The Effect of Herbal Medicine on Intestinal Flora and the Potential Molecular Mechanism. has shown some interesting foundlings on the herbal medicine with a profound effect on the intestinal flora, as well as the potential molecular mechanisms behind this phenomenon.
